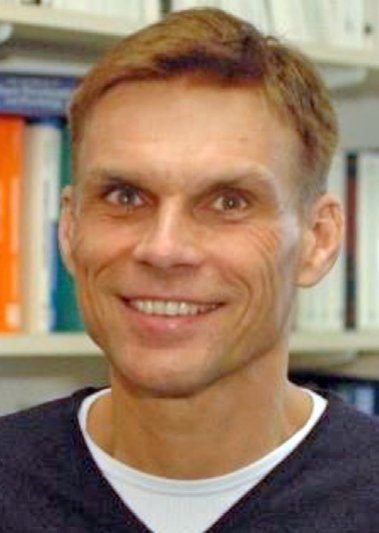# Radical‐Induced 1,2‐Migrations of Boron Ate Complexes

**DOI:** 10.1002/adsc.201901503

**Published:** 2020-02-21

**Authors:** Marvin Kischkewitz, Florian W. Friese, Armido Studer

**Affiliations:** ^1^ Organisch-Chemisches Institut Westfälische Wilhelms-Universität Corrensstrasse 40 48149 Münster Germany

**Keywords:** 1,2-boron ate rearrangements, boronic esters, electron catalysis, radical polar crossover reactions

## Abstract

1,2‐Boron ate rearrangements represent a fundamental class of transformations to establish new C−C bonds while retaining the valuable boron moiety in the product. In established ionic processes, the boron ate complex is activated by an external electrophile to induce a 1,2‐migration from boron to an adjacent *sp* ^3^ or *sp* ^2^ carbon atom. Recently, two complementary radical polar crossover approaches have been explored for both classes, 1,2‐migrations to *sp* ^2^ and *sp* ^3^ carbon centers. This review describes the general concepts in this emerging research field and summarizes recent developments of radical‐induced 1,2‐migrations from boron to carbon.

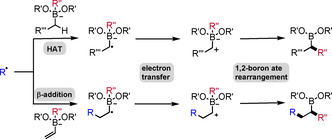

## 1 Introduction

**Scheme 1:**
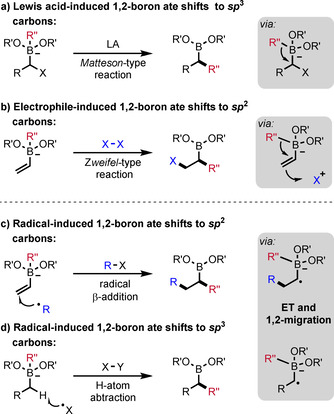
1,2‐Boron ate shifts to *sp* ^*2*^and *sp* ^*3*^carbons.

Organoboranes and boronic esters are highly valuable reagents to conduct various cross‐coupling reactions.^1^ Furthermore, the boron moiety can be converted into a broad range of functional groups, often with high levels of stereospecifity when considering chiral secondary or tertiary alkylboronic esters as substrates.^2^ Mechanistically, these transformations usually proceed by nucleophilic addition to the empty *p*‐orbital of the boron atom followed by a stereospecific 1,2‐migration to an adjacent electrophilic acceptor atom. In the early 1960s, Hillman^3^ and Matteson^4^ first demonstrated that 1,2‐boron ate rearrangements allow for C−C bond formation. Notably, in these transformations the valuable boron moiety remains in the product. Along these lines, the introduction of the air‐ and moisture‐stable boronic ester entity, in particular the pinacol boronic ester, opened a new field of organoboron homologation chemistry.^5^ As a first class of this important transformation type, 1,2‐boron ate shifts to *sp* ^3^ carbons bearing a suitable α‐leaving group have to be highlighted (Scheme 1a). In the so‐called Matteson reaction, which initiated the research field,^6^ a boron ate complex derived from a pre‐functionalized α‐halo boronic ester and an alkyl‐ or aryl‐metal compound is activated by a Lewis acid to induce the 1,2‐boron to carbon migration with concomitant substitution of the halide anion. Alternatively, the boron ate complex can be generated through the reaction of a boronic ester with a chiral carbanion bearing a suitable α‐leaving group (carbenoid‐type compound).^7^ In the second class, the π‐bond of a vinyl boron ate complex is activated by an external electrophile to trigger a 1,2‐shift to the former *sp* ^2^ α‐carbon center (Scheme 1b). The most prominent reaction of this class is the Zweifel olefination,^8^ in which the 1,2‐shift is induced by electrophilic halogenation of the vinyl moiety in the B‐ate complex. Following Murakami's studies^9^ on alkynyltrialkyl boron ates, Morken and co‐workers established in 2015 the use of transition metal complexes to activate alkenyl boron ate complexes in conjunctive cross‐couplings.^10^ Recently, two complementary radical approaches have been developed for both classes, 1,2‐migrations to *sp* ^2^ and *sp* ^3^ carbon centers.^11^ In the first approach, the 1,2‐boron to carbon migration is induced by an initial radical addition to the β‐position of a vinyl boron ate complex (Scheme 1c). In the second approach, a regioselective radical α‐C(*sp* ^3^)−H abstraction in a boron ate complex derived from a non‐pre‐functionalized alkyl boronic ester triggers the 1,2‐shift (Scheme 1d). In both cases a reducing radical anion^12^ is generated, which further reacts *via* electron transfer (ET) oxidation followed by 1,2‐aryl/alkyl migration. Of note, such radical polar crossover reactions usually occur *via* electron catalysis^13^ and proceed without the need of any transition metal catalyst. Published review articles in this field cover 1,2‐boron ate shifts that are triggered through ionic mechanisms.^5^, 6e, 6f, 7d, 8b In this perspective we will focus exclusively on radical‐induced 1,2‐migrations to *sp* ^2^ and *sp* ^3^ centers that have been discovered only recently.

## 2 Radical‐Induced 1,2‐Boron Ate Shifts to *sp* ^2^ Carbons

### 2.1 Vinyl Boron Ate Complexes

**Scheme 2:**
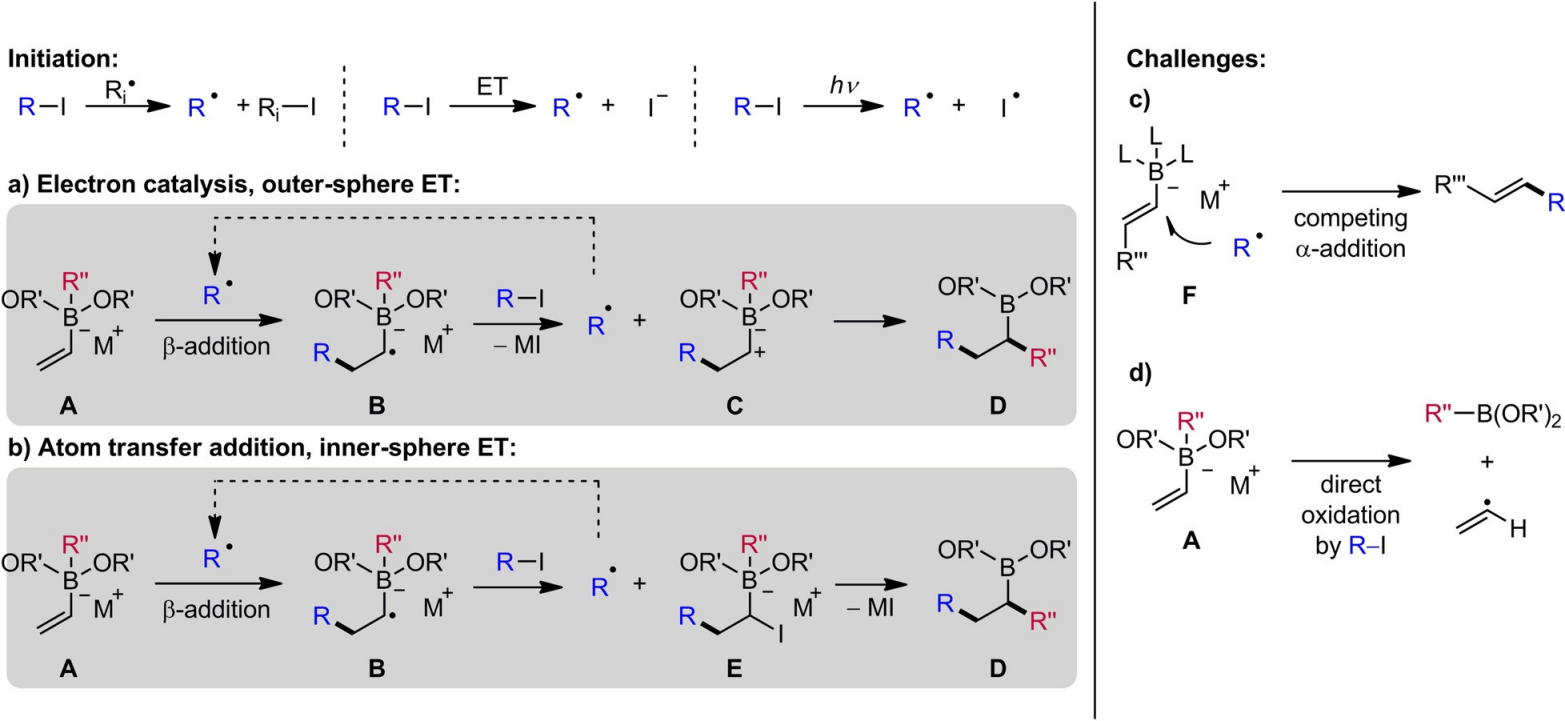
Reaction design and mechanistic considerations for radical‐induced 1,2‐migrations of vinyl boron ate complexes.

Vinyl boronic esters are highly important building blocks in organic synthesis.^14^ Such boron‐substituted alkenes have found attention as radical acceptors for more than 50 years,^15^ but the radical chemistry on the corresponding vinyl boron ate complexes has been explored only recently. In contrast to their neutral congeners, vinyl boron ate complexes bear an electron‐rich double bond and therefore express a high reactivity towards electrophilic alkyl radicals. The generation of such C radicals can be achieved from a radical precursor R−I by I abstraction, reductive mesolytic C−I cleavage or by photolytic homolysis of the C−I bond. Addition of the electrophilic alkyl radical R^.^ to the β‐position of the vinyl boron ate complex **A** generates the corresponding adduct radical anion **B** (Scheme 2a). In the following radical polar crossover step, adduct **B** undergoes an outer‐sphere electron transfer oxidation to deliver zwitterion **C**. According to an electron‐catalyzed process,^13^ the electron is directly transferred to R−I, thereby generating radical R^.^ sustaining the chain. In the subsequent ionic 1,2‐R′′‐migration the carbon substituent is transferred from boron to the α‐carbon atom. The overall process comprises the formation of two C−C bonds and delivers a boronic ester of type **D**. An alternative inner‐sphere electron transfer mechanism is presented in Scheme 2b. In this scenario, adduct radical anion **B** could abstract the I atom of R−I to give the atom transfer product **E**, which could further engage in a Matteson‐type 1,2‐R′′‐migration to deliver the same target **D**. Whether the reaction proceeds by outer‐sphere (**B** to **C**) or inner‐sphere (**B** to **E**) electron transfer depends on the reduction potential of the alkyl radical precursor and on the halogen atom transfer efficiency.13b However, this reaction design causes two major challenges. Firstly, especially β‐substituted vinyl boron ate complexes are prone to undergo a competing radical α‐addition leading to the formation of *trans*‐alkenes (Scheme 2c). This reactivity was established by the groups of Buchwald and Akita for the transformation of vinyltrifluoro boron ate complexes with the Togni reagent^16^ in the presence of Fe(II) salts^17^ or an Ru‐photoredox catalyst.^18^ Later, that approach was further extended by Leonori and co‐workers to the coupling of vinyl boron ate compounds with various electrophilic alkyl radicals.^19^ Secondly, the alkyl radical precursor should be a mild oxidant that does not directly react with the starting electron‐rich boron ate complex *via* an electron transfer process leading to the formation of the ethenyl radical (Scheme 2d).

**Scheme 3:**
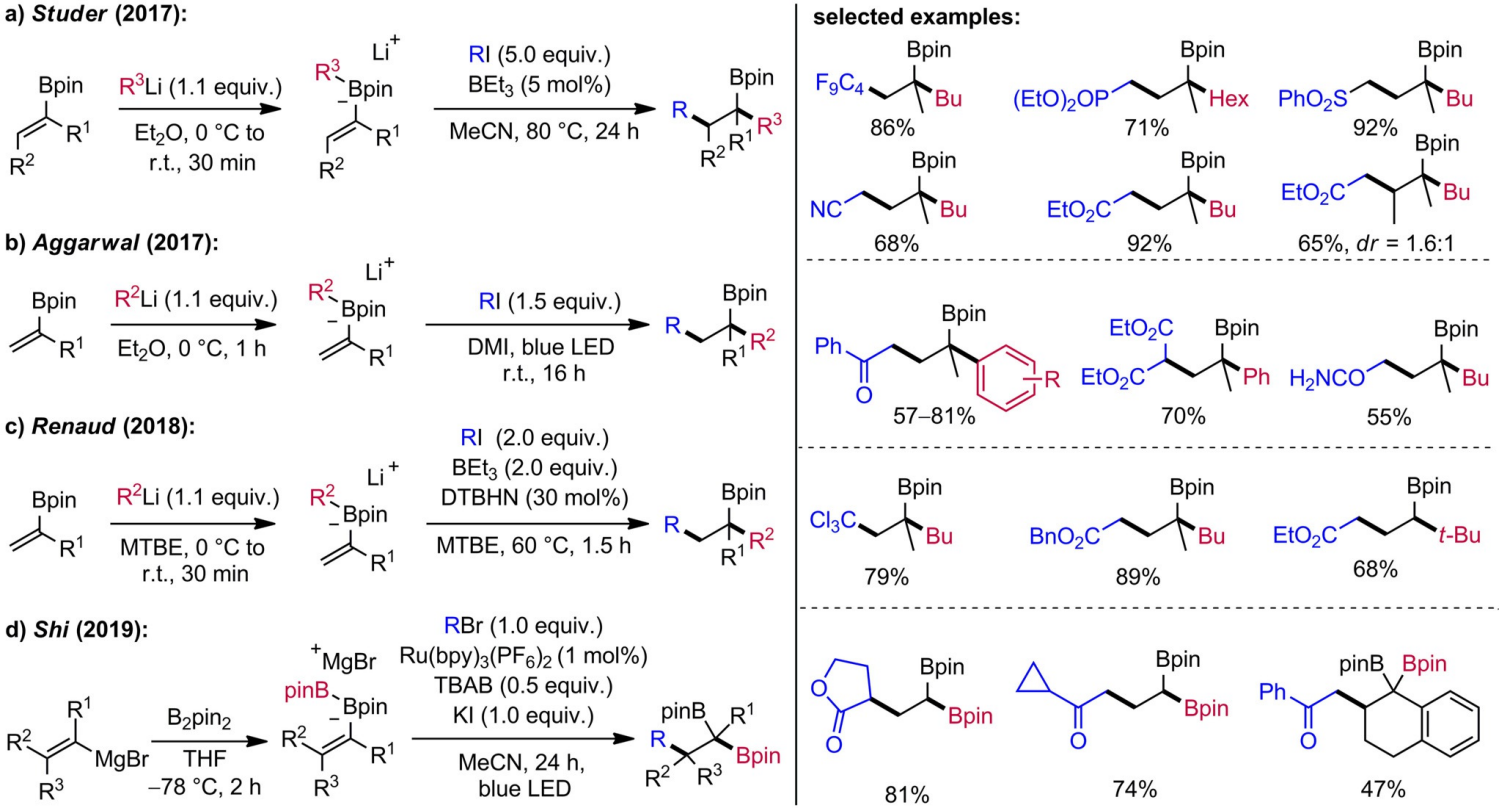
Radical‐induced 1,2‐migrations of vinyl boron ate complexes: synthesis of functionalized secondary and tertiary alkyl boronic esters.

In 2017, the groups of Studer and Aggarwal independently implemented such a strategy (Scheme 3a, b). In both methods the sequence commences with the *in situ* formation of the vinyl boron ate complex by treatment of a vinyl boronic pinacol ester with an aryl‐ or alkyl‐lithium compound. After solvent exchange, the boron ate complex can be used in the radical sequence without any further purification. Notably, the three‐component coupling tolerates α‐ and β‐substituted vinyl boron ate complexes and the substrate scope with respect to the radical precursor R−I includes perfluoroalkyl iodides, α‐iodo sulfonates, α‐iodo phosphonates, α‐iodo nitriles, α‐iodo esters, α‐iodo ketones, primary α‐iodo amides and diethyl bromomalonates allowing the rapid build‐up of molecular complexity. In the Studer protocol, initiation of the radical chain reaction is achieved with BEt_3_/O_2_ (Scheme 3a).^20^ In contrast, the Aggarwal approach initiation proceeds under blue light irradiation (Scheme 3b).^21^ Moreover, less reactive alkyl bromides could successfully be employed in the presence of 20 mol% sodium iodide or 1 mol% of a smart Ru‐photoinitiator. The term smart initiation was first introduced by Studer and Curran to describe catalysis of initiation and is especially valuable if short chains are involved. In the context of this article, a smart initiator is interpreted as a redox‐active compound that is eligible to both reductively initiate a radical chain reaction and to be regenerated by oxidation of a reducing species of the innate chain (in this case radical anion **B**, see Scheme 2). Even though in boron ate chemistry so far only photoredox catalysts have been described as smart initiators, the concept is readily applicable to other substance classes. One obvious feature of smart initiation is displayed by the quantum yield Φ, which is often determined to be greater than 1. Consequently, one photon generates multiple product molecules, strongly suggesting that an innate radical chain is operative and the photoredox catalyst is mainly involved in the initiation step.13b

The practical value of the Studer protocol was further documented in an improved and experimentally checked *Organic Synthesis* procedure using only 1.5 equivalents of the radical precursor under irradiation with visible light.^22^ In 2018, Renaud and co‐workers presented a related protocol that can be performed without solvent exchange in methyl *tert*‐butyl ether (MTBE). Nevertheless, 2.0 equivalents of BEt_3_ and 30 mol% di‐*tert*‐butyl hypodinitrite (DTBHN) are required for initiation (Scheme 3c).^23^ In additional mechanistic experiments, the authors could further support that the mechanism likely proceeds *via* an outer‐sphere electron transfer process (see Scheme 2a). More recently, Shi and co‐workers demonstrated that *in situ* generated alkenyl diboron ate complexes derived from alkenyl‐Grignard reagents and bis(pinacolato)diboron undergo efficient radical‐induced 1,2‐boron to carbon shifts of a boron group to give various *gem*‐bis(boryl)alkanes (Scheme 3d).^24^ Of note, the quantum yield of this process was determined to be Φ=49.8, clearly showing that the Ru complex mainly acts to initiate an innate radical chain reaction.

**Scheme 4:**
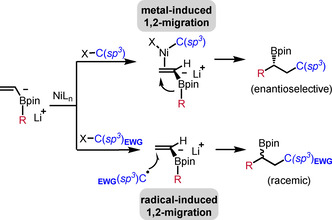
Radical‐ionic mechanistic dichotomy.

In 2017, Lovinger and Morken disclosed their results on a Ni‐catalyzed enantioselective conjunctive coupling with C(*sp* ^3^) electrophiles.^25^ Interestingly, for non‐activated alkyl halides the authors observed high enantioselectivity, whereas the reactions with the electron‐poor congeners furnished racemic products (Scheme 4). Mechanistic experiments revealed that the origin of this different reaction outcome is a radical‐ionic mechanistic dichotomy. In the case of non‐activated alkyl halides, the vinyl boron ate complex engages in an enantioselective metal‐induced 1,2‐boron ate shift with subsequent reductive elimination in analogy to the previously described Pd‐catalyzed enantioselective conjunctive couplings10a with C(*sp* ^2^) electrophiles. In contrast, activated C(*sp* ^3^) electrophiles are reduced more easily and an Ni‐initiated radical polar crossover chain reaction is operative.

Although a proof of principle experiment for a stereoselective radical‐induced 1,2‐migration provided a promising 52% enantiomeric excess by using a chiral vinyl boronic acid pinanediol ester in combination with phenyllithium and perfluorobutyl iodide,^20^ a general enantioselective three‐component coupling strategy for electron‐poor alkyl iodides as alkyl radical precursors still remains to be developed.

**Scheme 5:**
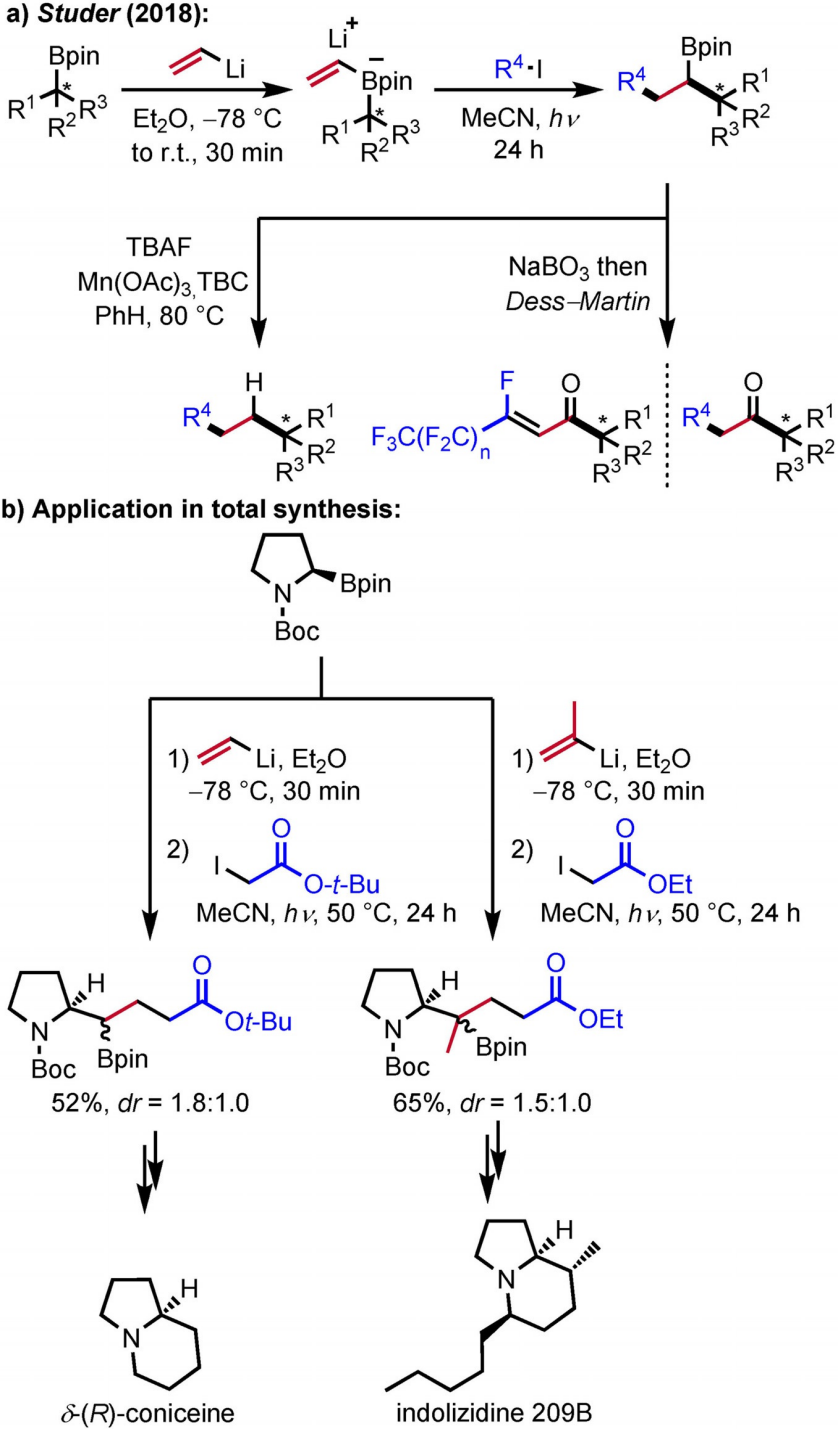
Stereospecific radical‐polar crossover reaction of chiral boronic esters and its application to the synthesis of *δ*‐(*R*)‐coniceine and indolizidine 209B.

Highly enantioenriched compounds are readily accessible by the radical polar crossover strategy using chiral alkyl boronic esters as substrates for the formation of the corresponding vinyl boron ate complexes. Along these lines, the radical‐induced 1,2‐boron ate rearrangement was successfully used for the preparation of α‐chiral ketones and enantioenriched alkanes, which were obtained after oxidation of the initial B‐containing products with sodium perborate and Dess–Martin periodinane or by protodeborylation, respectively (Scheme 5a).^26^ In these processes, vinyl boron ate complexes derived from enantioenriched alkyl boronic esters and vinyllithium are reacted with various commercially available alkyl iodides. Notably, chiral boronic esters are easily accessible by hydroboration,^27^ the Matteson approach (substrate control)^6^ or by lithiation–borylation using Hoppe's chiral lithiated carbamates (reagent control).^7^ Deborylative follow‐up chemistry was necessary, since the stereocenter at the α‐position to the boron atom could not be controlled in the radical polar crossover cascade. If α‐perfluoroalkylated ketones are formed as initial products after C−B to C=O functional group transformation, HF elimination was observed during purification leading to α,β‐unsaturated ketones. The high synthetic potential of the stereospecific radical‐induced 1,2‐boron ate rearrangement was further demonstrated by its application to novel formal total syntheses of δ‐(*R*)‐coniceine and indolizidine 209B, starting with the enantiopure Boc‐protected pyrrolidine boronic ester (Scheme 5b).^28^ *Via* a radical polar crossover reaction using α‐iodoacetates as C‐radical precursors, two C−C‐bonds are formed in the key reaction. A sequence of lactamization, protodeborylation and reduction of the lactams finally gives the desired natural products.

### 2.2 Dienyl Boron Ate Complexes

**Scheme 6:**
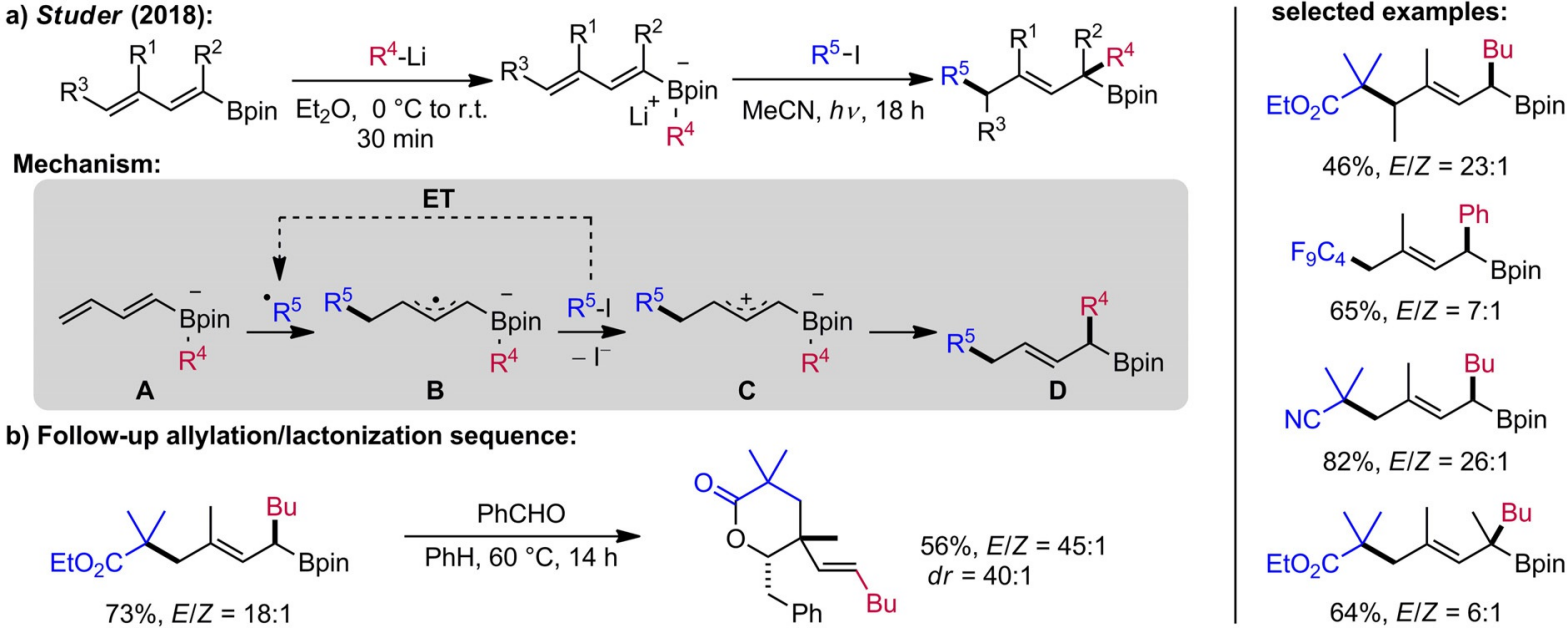
Radical‐polar crossover reaction of dienyl boron ate complexes.

Inspired by the radical polar crossover reaction of “simple” vinyl boron ate complexes (see Section 2.1), the Studer group investigated dienyl boron ate complexes as radical acceptors.^29^ If the addition of the transient C‐radical selectively occurs at the δ‐position of the boron ate complex **A**, an allyl radical intermediate **B** is generated. Triggered by electron‐transfer oxidation, it was assumed that **B** might then undergo a 1,2‐boron ate rearrangement *via* zwitterionic intermediate **C** to give allyl boronic esters of type **D**, thereby propagating the chain reaction by generation of the transient C‐radical R^5.^. In fact, this scenario was realized using various boron ate complexes derived from dienyl boronic esters and aryl‐ or alkyllithium reagents in combination with electron‐poor alkyl iodides under visible light irradiation (Scheme 6a). The geometry of the double bond arising from reactions of the alkenyl boron ate complexes in Scheme 6 was well controlled in most cases with the *E*‐isomer being formed with good to excellent selectivity. Notably, side products formed by the undesired β‐addition to the ate complex **A** were not detected, revealing the high intrinsic δ‐selectivity of the dienyl boron ate acceptor. The allyl boronic ester products are valuable building blocks in synthesis and were employed in an allylation/lactonization sequence to afford highly substituted δ‐lactones with excellent diastereoselectivity as exemplified in Scheme 6b.

### 2.3 Heteroaryl Boron Ate Complexes

**Scheme 7:**
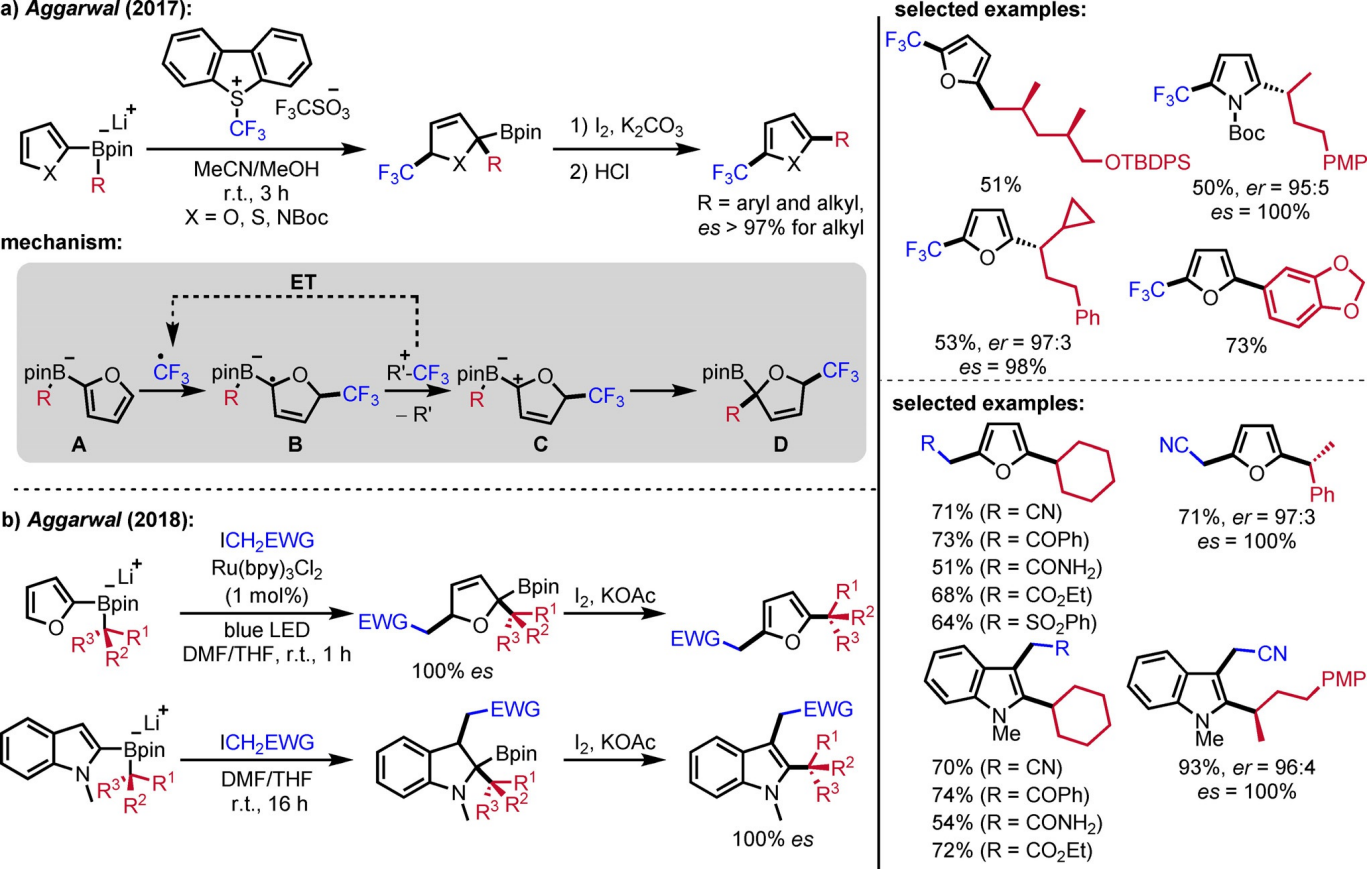
Radical‐induced three‐component coupling of heteroarenes with boronic esters.

Based on previously reported electrophile‐induced enantiospecific coupling of alkyl boronic esters with lithiated electron‐rich arenes,^30^ Aggarwal disclosed a trifluoromethyl‐radical induced three‐component coupling of boron ate complexes derived from boronic esters and lithiated furans (Scheme 7a).^31^ In this elegant process, the Umemoto reagent is used as the trifluoromethyl radical precursor and the chain‐carrying trifluoromethyl radical regioselectively adds to the electron‐rich furan moiety of the boron ate complex **A** to give the corresponding radical anion **B**. Electron transfer from intermediate **B** to the Umemoto reagent generates the trifluoromethyl radical along with the zwitterionic species **C** that rapidly undergoes a 1,2‐boron ate rearrangement to provide the substituted dihydrofuran **D**. Such furans are isolable but can also be readily converted to the corresponding 2,5‐functionalized furans upon treatment with iodine under basic conditions. In the overall sequence, the pharmacologically relevant CF_3_‐group along with an aryl or an alkyl group can be transferred to the furan moiety, the latter with complete enantiospecifity. Later, this protocol was extended to electron‐poor alkyl iodides as substitutes for the Umemoto reagent and chain initiation was achieved by irradiation with blue LEDs (Scheme 7).^32^ This required addition of Ru(bpy)_3_Cl_2_ to the reaction mixture, acting as a smart initiator – a high quantum yield of well above 1 (Φ=27.8) suggests a radical chain mechanism to be operative in these cascades.13b For indoles, neither irradiation nor a photoredox catalyst was required to achieve product formation. Based on mechanistic studies, the authors stated that, in this particular case, the reaction likely follows a non‐radical S_N_2 pathway.^32^

## 3 Radical‐Induced 1,2‐Boron Ate Shifts to *sp* ^3^ Carbons

### 3.1 Radical Addition to Bicyclobutyl Boron Ate Complexes

**Scheme 8:**
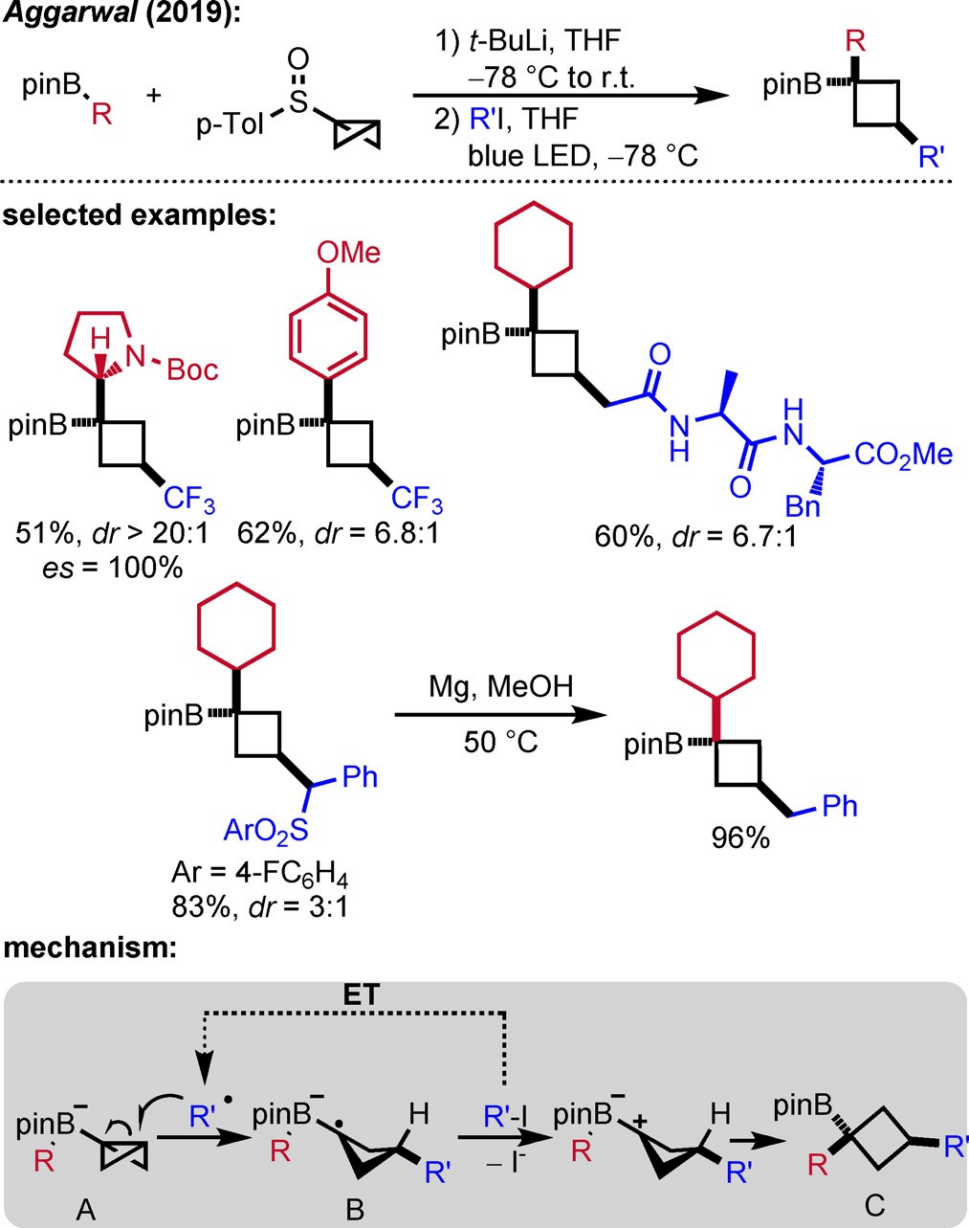
Radical‐induced 1,2‐boron ate rearrangement of BCB boron ate complexes.

While the generation of *sp* ^3^‐centered radicals *via* radical addition to π‐systems is a common approach in synthetic chemistry, radical generation *via* homolytic substitution at carbon (*sp* ^3^) is not well established. Due to the high intrinsic strain energy and the partial π‐character of the central C−C bond of a bicyclobutane (BCB), such a moiety can act as a radical acceptor.^33^ Aggarwal first employed BCB boron ate complexes of type **A** in a radical polar crossover sequence (Scheme 8).^34^ The required BCB boron ate complex **A** is accessible *via* lithiation of BCB *p*‐tolyl sulfoxide with *t‐*BuLi and subsequent addition to a boronic ester.^35^ Upon radical addition, the ring strain is released, leading to a cyclobutyl radical intermediate **B**, which can further react *via* a 1,2‐boron ate rearrangement triggered by oxidation to finally give a functionalized cyclobutane **C**. This structural motif has received growing interest in medicinal chemistry.^36^ The strongly reducing properties of the boryl radical anion **B** allowed the use of various electron‐deficient alkyl iodides such as perfluoroalkyl iodides, α‐iodomethyl sulfonates, α‐iodo nitriles, α‐iodo acetates and α‐iodo amides as C‐radical precursors. Non‐activated alkyl iodides were found to be inactive. Notably, both the radical addition and the subsequent migration are stereochemically well controlled leading to a *cis*‐configuration in the products. For most substrates, good to excellent diastereoselectivity was found and the migration occurred with complete stereospecifity, as expected.

### 3.2 H‐Transfer‐Induced Coupling of Alkylboronic Esters and Organometallic Reagents

Intermolecular HAT is a valuable strategy for the direct functionalization of C(*sp* ^3^)−H bonds, albeit sometimes difficult to control. Although this approach for C−H functionalization has been known for decades, most preparatively useful protocols in this field have appeared in the last ten years in the context of photoredox catalysis.^37^, ^38^ In contrast to heteroatom‐centered radicals, C‐radicals usually lack thermodynamic driving force to engage as H‐acceptors in intermolecular HATs. The highly reactive CF_3_ radical is an exception along those lines.^39^

**Scheme 9:**
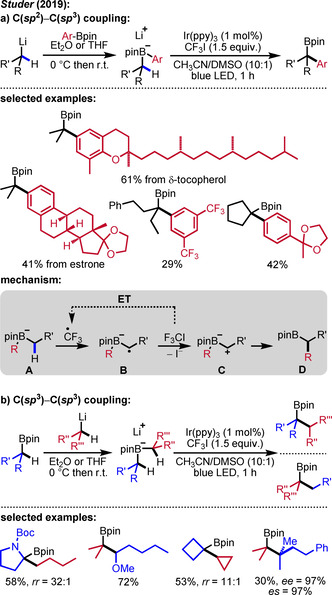
HAT‐induced coupling of alkyl boronic esters with organometallic reagents.

The Studer group recently disclosed highly regioselective generation of *sp* ^3^‐centered carbon radicals *via* intermolecular HAT from alkyl boron ate complexes to CF_3_ radicals (Scheme 9).^40^ Instead of the α‐pre‐functionalized organometallic reagents used in the classical Matteson reaction, unfunctionalized alkyl boronic esters can be applied as substrates. In these transformations, the target α‐C−H bond of the boron ate complex is directly activated to trigger the 1,2‐boron ate rearrangement, thus significantly increasing step economy. In the key step, a hydrogen atom is transferred from the α‐C(*sp* ^3^)−H bond of a previously formed boron ate complex **A** to a reactive CF_3_ radical. Subsequent oxidation of the intermediately generated radical **B** to the zwitterionic species **C** induces a 1,2‐boron ate rearrangement to afford the desired rearranged product **D**. CF_3_I is used as the oxidant and in the oxidation of radical **B**, the chain‐carrying trifluoromethyl radical is generated along with **C**. One obvious challenge in this sequence is the realization of a regioselective HAT, where the trifluoromethyl radical turns out to be ideally suited as active species. Likely, polar effects play a key role in the selective α‐H‐abstraction of these anionic boron ate complexes by the electrophilic CF_3_ radical (see below). Moreover, it is well established that CF_3_I is an excellent terminal oxidant for intermediates of type **B**,^20^, ^26^, ^29^ rendering this cheap iodide to be the reagent of choice to realize this interesting sequence.^39^ Ir(ppy)_3_ was employed as a smart initiator upon irradiation with blue LEDs (Φ=8.8). In selected cases, initiation was achieved with blue LED irradiation in the absence of the Ir complex, underlining the apparent chain‐type reaction mechanism.13b This methodology enables both the α‐C−H arylation (Scheme 9a) and α‐C−H alkylation of alkylboronic esters (Scheme 9b). Considering the latter transformation, the starting dialkyl boron ate complexes offer two different C−H sites at the α‐position to boron that have to be differentiated by the CF_3_ radical. It was found that HAT selectivity follows a clear trend in which generally the weaker and less sterically hindered C−H bond is preferably activated. Interestingly, the isopropyl group out‐competes the α‐methoxyalkyl group, emphasizing that conformation likely also plays a crucial role in determining the regioselectivity of the intermolecular HAT. Due to the stereospecific nature of the 1,2‐boron ate rearrangement, enantioenriched boronic esters are also accessible *via* the radical induced α‐C−H alkylation reaction (Scheme 9b). The key HAT transfer step was further investigated by density functional theory (DFT) calculations. These theoretical studies revealed that the high HAT selectivity is caused by polar effects leading to low energy barriers for the transfer of the α‐H‐atom of the boron ate complex to the electrophilic CF_3_ radical.

## 4 Summary and Outlook

In recent years, radical chemistry has experienced a renaissance and accordingly also boron‐based radical chemistry has matured. Various methods for C‐radical borylation to access boronic esters have been developed.^41^ In these reactions, the boron moiety gets introduced into an organic compound. There are also radical transformations in which the boronic esters are used as substrates. For example, vinyl boronic esters have been shown to react as acceptors with various C‐radicals with pioneering studies being published decades ago. In contrast, radical transformations on boron ate complexes represents a very young research field that has evolved during the last two years. As discussed in this perspective, in radical cascades involving boron ate complexes, the radical bond‐forming step can be combined with an ionic 1,2‐metallate rearrangement from boron to carbon culminating in a radical polar crossover process. This is meanwhile well documented for C‐radical addition to vinyl boron ate complexes. Since ionic 1,2‐boron to carbon migrations of alkyl groups are stereospecific, such crossover chemistry allows the preparation of enantiomerically pure compounds. Alkyl, aryl and even boryl groups can act as the migrating moieties in these 1,2‐rearrangements. Such cascades are usually conducted under mild conditions, tolerate many functional groups and are highly modular. The boron moiety remains in the product and thus such compounds can serve as valuable substrates for follow‐up chemistry.

Despite great recent achievements in this emerging research area, some limitations have become apparent, especially considering enantioselective radical polar crossover processes. In addition, current cascades are restricted to a rather small set of C‐radicals. For example, heteroatom‐centered radicals have not yet been used in combination with boron ate complexes as reaction partners. To further expand the synthetic utility of the radical‐induced 1,2‐boron ate rearrangement, electrochemistry or extended combination with transition metal catalysis might provide answers. In any case, the authors of this perspective are confident that many exciting findings will be published in this highly active research field in future.

## Biographical Information

*Marvin Kischkewitz* was born in Essen, Germany, in 1990. He received his BSc and MSc degrees in chemistry from the Westfälische Wilhelms‐Universität Münster, Germany. After a research stay in the laboratory of Professor Mark Lautens at the University of Toronto he joined the group of Professor Armido Studer in Münster, where he completed his PhD in 2019. His research focuses on boron‐based radical reactions.

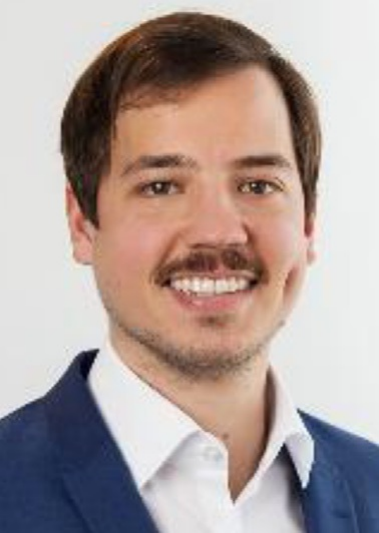

## Biographical Information

*Florian W. Friese* was born in Hamm, Germany, in 1990. He received his MSc in chemistry from the Westfälische Wilhelms‐University (Münster, Germany) under the supervision of Prof. Günter Haufe in 2015. He then joined the group of Prof. Armido Studer and finished his PhD in 2019. His work mainly focuses on the development of new radical chain reactions for the functionalization of aliphatic alcohols.

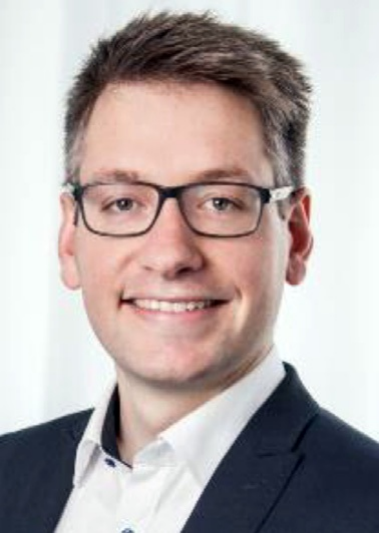

## Biographical Information

*Armido Studer* received his Diploma in 1991 and his PhD in 1995 from ETH Zürich under the direction of Prof. Dieter Seebach. He continued his scientific education as a Swiss National Science Foundation Post‐doctoral Fellow at the University of Pittsburgh with Prof. Curran. In 1996 he started his independent career at the ETH Zürich. In 2000, he was appointed as associate professor at the Philipps‐University in Marburg, Germany and in 2004 as full professor of organic chemistry at the Westfälische Wilhelms‐University in Münster, Germany. His research interests focus on the development of new synthetic methods. In addition, he also works on living radical polymerizations, on the preparation of functional polymers and on the development of methods for the chemical modification of surfaces.